# Functional Characterization of Alternative and Classical Pathway C3/C5 Convertase Activity and Inhibition Using Purified Models

**DOI:** 10.3389/fimmu.2018.01691

**Published:** 2018-07-23

**Authors:** Seline A. Zwarthoff, Evelien T. M. Berends, Sanne Mol, Maartje Ruyken, Piet C. Aerts, Mihály Józsi, Carla J. C. de Haas, Suzan H. M. Rooijakkers, Ronald D. Gorham

**Affiliations:** ^1^Department of Medical Microbiology, University Medical Center Utrecht, Utrecht University, Utrecht, Netherlands; ^2^Department of Immunology, ELTE Eötvös Loránd University, Budapest, Hungary

**Keywords:** innate immunity, inflammatory disease, convertase enzymes, complement, complement therapeutics, multi-molecular proteases

## Abstract

Complement is essential for the protection against infections; however, dysregulation of complement activation can cause onset and progression of numerous inflammatory diseases. Convertase enzymes play a central role in complement activation and produce the key mediators of complement: C3 convertases cleave C3 to generate chemoattractant C3a and label target cells with C3b, which promotes phagocytosis; C5 convertases cleave C5 into chemoattractant C5a, and C5b, which drives formation of the membrane attack complex. Since convertases mediate nearly all complement effector functions, they are ideal targets for therapeutic complement inhibition. A unique feature of convertases is their covalent attachment to target cells, which effectively confines complement activation to the cell surface. However, surface localization precludes detailed analysis of convertase activation and inhibition. In our previous work, we developed a model system to form purified alternative pathway (AP) C5 convertases on C3b-coated beads and quantify C5 conversion *via* functional analysis of released C5a. Here, we developed a C3aR cell reporter system that enables functional discrimination between C3 and C5 convertases. By regulating the C3b density on the bead surface, we observe that high C3b densities are important for conversion of C5, but not C3, by AP convertases. Screening of well-characterized complement-binding molecules revealed that differential inhibition of AP C3 convertases (C3bBb) and C5 convertases [C3bBb(C3b)_n_] is possible. Although both convertases contain C3b, the C3b-binding molecules Efb-C/Ecb and FHR5 specifically inhibit C5 conversion. Furthermore, using a new classical pathway convertase model, we show that these C3b-binding proteins not only block AP C3/C5 convertases but also inhibit formation of a functional classical pathway C5 convertase under well-defined conditions. Our models enable functional characterization of purified convertase enzymes and provide a platform for the identification and development of specific convertase inhibitors for treatment of complement-mediated disorders.

## Introduction

The human complement system comprises a family of proteins that are essential to the human immune response against infections (1). Complement recognizes microbes or damaged host cells and subsequently triggers an enzymatic cascade that mainly serves to (a) label target cells for phagocytosis by immune cells, (b) produce chemoattractants, and (c) directly kill target cells *via* pore formation (2). Unwanted complement activation on the body’s own cells is a key pathological driver in a wide spectrum of immune diseases including autoimmune, inflammatory, and degenerative diseases (3–5). For current and future development of therapeutic complement inhibitors, knowledge of complement activation and how it can be regulated is of great importance.

**Figure 1:**
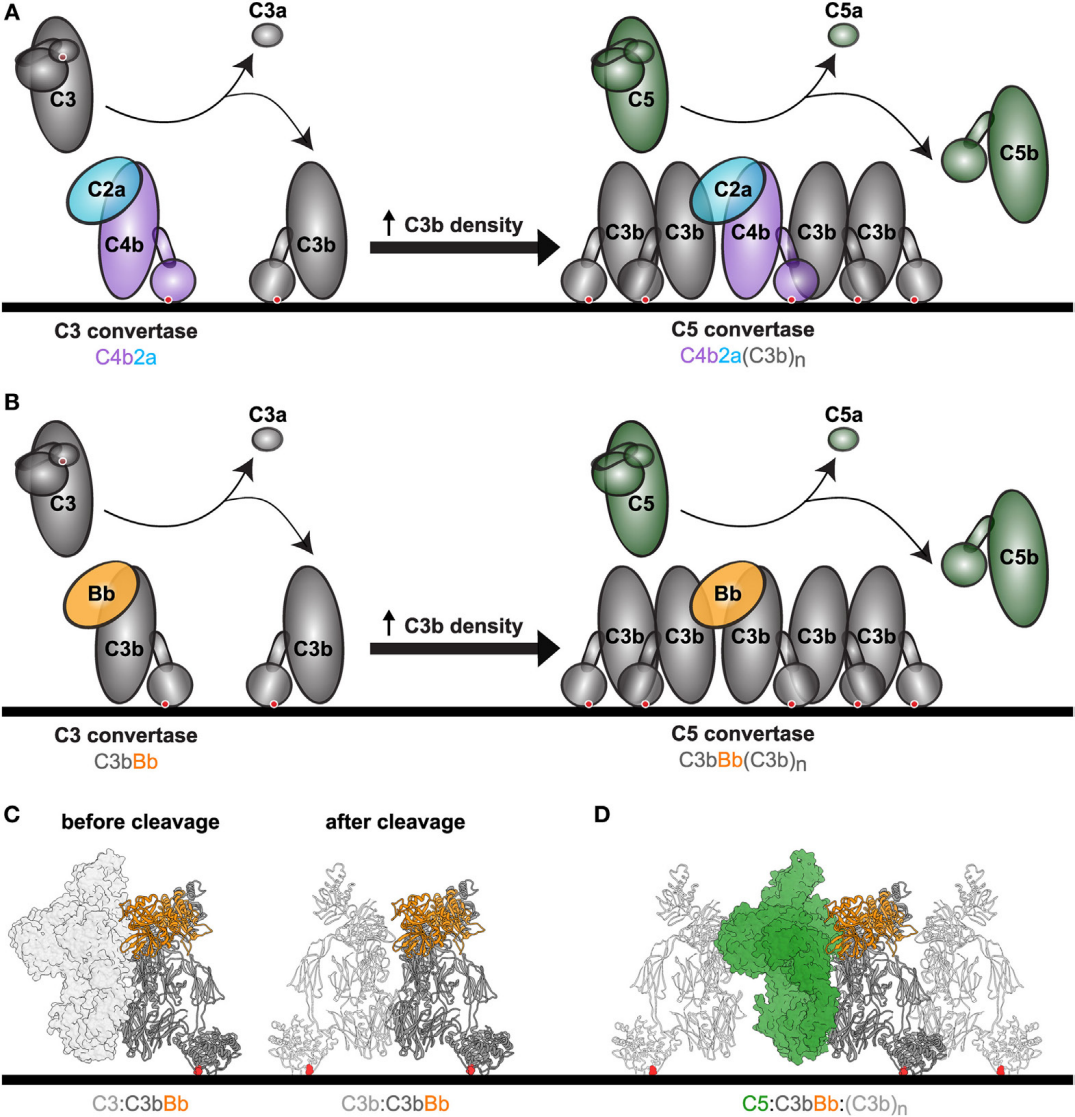
Complement convertases mediate C3 and C5 conversion. **(A)** Upon complement activation, C3 convertases consisting of either C4b2a (CP and LP) or **(B)** C3bBb [alternative pathway (AP)] form on the cell surface. Conversion of C3 results in deposition of C3b molecules *via* the thioester (red dot), which form the basis for new AP convertases (amplification loop) or associate with existing C3 convertases to form C5 convertases. These accessory C3b molecules (C3b_n_) enable efficient C5 conversion, however, the molecular mechanisms underlying this process are not clear. Shown are C4b (purple), C2a (blue), C3 and cleavage products C3b/C3a (gray), Factor B (orange), C5 and cleavage products C5b/C5a (green). **(C)** Structural models of the C3 convertase C3bBb with its substrate C3 before and after cleavage. Models are based on structures of the C3bBb-SCIN dimer (PDB: 2WIN). The convertase is shown in ribbon representation, with C3b in dark gray and Bb in orange. On the left, the substrate C3 (light gray surface) is shown before cleavage. On the right, the product C3b (light gray licorice) is shown after cleavage. The red dots highlight the thioester. **(D)** Structural model of the previously proposed AP C5 convertase with its substrate C5. At a high density of C3b molecules, C5 is recruited to the target surface and can be cleaved after binding of Bb to C3b. The exact molecular arrangement of the C5 convertases remains unknown. This structural model is based on the CVF-C5 crystal structure (PDB: 3PVM), with accessory C3b molecules added manually surrounding the convertase and C5. CVF (cobra venom factor) is a C3b homolog that lacks the thioester domain and forms stable C5 convertases when associated with Bb in solution. Structure is shown in ribbon representation with C3b (dark gray), Bb (orange), accessory C3b molecules (light gray licorice), and C5 (green surface).

Convertase enzymes fulfill a central role in the complement cascade as they cleave C3 and C5, which mediate nearly all complement effector functions. C3 convertases cleave C3 into C3a, a chemoattractant molecule, and C3b, which covalently binds to target surfaces and triggers phagocytosis. C5 convertases cleave C5 into C5a, a potent mediator of leukocyte recruitment and inflammation, and C5b, the initiator of the membrane attack complex and cell lysis. The complement cascade begins *via* specific recognition of target cells in the classical (CP) and lectin (LP) pathways. In the CP, antibodies bind epitopes on the target cell and subsequently recruit the C1 complex (C1qr_2_s_2_). Upon binding to the antibody platforms (6), C1q-associated protease C1s converts C4 and C2 to generate a C3 convertase enzyme (C4b2a) on the cell surface (Figure 1A). Similarly, the lectin pathway also forms C4b2a *via* activation of mannose-binding lectin-associated serine proteases. The resulting CP/LP C4b2a convertases cleave C3 into C3a and C3b. Following cleavage, a reactive thioester in C3b is exposed, which enables its covalent attachment to target cell surfaces, leading to recognition of the cells by phagocytes. The labeling of target cells with C3b is amplified by the alternative pathway (AP) in which surface-bound C3b binds factor B (FB). The proconvertase C3bB is then cleaved by factor D (FD) to form an active C3 convertase complex that consists of C3b and the protease fragment Bb (C3bBb) (Figure 1B). Since the resulting active AP C3 convertase (C3bBb) is comprised of C3b itself, substrate cleavage results in generation of additional convertases, further propagating C3b deposition (Figure 1B). When the density of C3b molecules on the cell surface becomes sufficiently high, the existing C3 convertases (C4b2a and C3bBb) gain the ability to cleave C5, leading to formation of C5a and C5b (Figures 1A,B) (7, 8).

Selective inhibition of C3 and C5 convertases is of great therapeutic interest. Most complement inhibitors currently used in the clinic or in clinical development target precursor (not yet activated) complement proteins, that circulate through the body and do not mediate complement effector functions (4). Due to high concentrations of these precursor proteins, effective therapeutic concentrations of complement inhibitors are often quite high, and clearance of these molecules is enhanced due to rapid turnover of complement proteins. Furthermore, saturation of precursor proteins is more likely to systemically suppress complement activation, leading to increased susceptibility to infection (4). Such therapies would be more effective if they specifically targeted active protein complexes like convertases that are primarily formed during complement activation on target cell surfaces. While some therapeutic molecules inhibit convertase function, these likely inhibit multiple convertase enzymes and block all effector functions of complement (4). In some cases, specific inhibition of C5 convertases is desirable for complement therapy, since blocking these would prevent unwanted formation of the major inflammatory trigger C5a but leave C3b deposition *via* C3 convertases intact and thus phagocytosis of bacteria. However, the molecular details of C5 convertase formation and C5 cleavage remain poorly understood, largely due to the transient nature of convertases and the fact that efficient C5 conversion is constrained to cell surfaces (7, 9). Several earlier studies successfully investigated individual convertases in purified or semi-purified environments (10–14), however, no single model can fully characterize activity and inhibition of both AP and CP C3 and C5 convertases in a purified and controlled environment. Herein, we extended our recently developed model system for AP C5 convertases (7) to also study surface-bound AP C3 convertases, in order to screen for specific inhibitors of convertase enzymes. Furthermore, we developed functional analyses to study CP C3 and C5 convertases using purified complement proteins. Using these models, we can evaluate how known complement inhibitors affect C3/C5 conversion. In these analyses, we included bacterial and therapeutic complement inhibitors, and human complement regulatory proteins that protect healthy tissue from complement attack (8, 15–18). The analyses reveal that several C3b-binding molecules can discriminate between C3 and C5 convertases, suggesting that it is possible to develop more specific convertase inhibitors in the future. Through comparison of our inhibitory data with previously reported structural and biochemical data, we further postulate molecular models of convertase formation.

## Materials and Methods

### Complement Proteins

C3 and C5 were prepared from human plasma as previously described (7). For CP assays and inhibitor dose-response assays, recombinant C5 was used. C5 with a C-terminal His-tag was therefore cloned from gBlocks (Integrated DNA Technologies) using Gibson assembly (Gibco), expressed in HEK293 cells (U-Protein Express, Utrecht, The Netherlands) and purified on a HisTrap column (GE Healthcare). C3b_H2O_ and C3b-PEG11-biotin were prepared as previously described using 180 µg/ml maleimide-PEG11-biotin for the latter (Thermo Scientific Pierce Protein Research, IK, USA) (7). Methylamine-treated C3 (C3_MA_) was prepared by mixing 2.7 µM C3 with 300 mM methylamine hydrochloride (Sigma Aldrich) in VBS^++^ buffer (Veronal Buffered Saline pH 7.4, 0.25 mM MgCl_2_, 0.5 mM CaCl_2_). This reaction was incubated at 37°C for 1 h and dialyzed overnight to VBS^++^ buffer at 4°C. FB with N-terminal His-tag and FD were expressed recombinantly as previously described (U-Protein Express, Utrecht, The Netherlands) (7). C4 was isolated from blood from a healthy individual that was anti-coagulated with 20 mM EDTA. Plasma was collected and protease inhibitors (10 mM benzamidine, 1 mM PMSF, 7.5 µM SBTI, EDTA 5 mM, 2.1 mM Pefabloc SC, 30 µM NPGB) were added quickly, while stirring the plasma at 4°C. To remove large complexes, plasma was precipitated with 4% PEG 6000, which was added slowly to the plasma for 45 min. After centrifugation, the supernatant was isolated from which plasminogen was removed by adding 20 mM EDTA and Lysine-Sepharose and incubation for 1 h at 4°C. From the supernatant, C4 was isolated by SourceQ anion exchange. Loading was performed in 50 mM Tris–HCl, 100 mM NaCl, pH 8.0 (containing 1 mM benzamidine, 1 mM PMSF, 30 mM EACA, and 5 mM EDTA) after which C4 was eluted in a gradient of 100–500 mM NaCl in 50 mM Tris–HCl, 100 mM NaCl, pH 8.0 (containing 1 mM benzamidine, 1 mM PMSF, 30 mM EACA, and 5 mM EDTA). Fractions were analyzed by 10% SDS-PAGE following Instant Blue (Roche) protein staining according to the manufacturer’s instructions. C2 with a N-terminal His-tag was expressed in HEK293 cells stably expressing EBNA1 (HEK293E) as described (U-Protein Express, Utrecht, The Netherlands) (19). C2 was purified from expression medium *via* immobilized metal affinity chromatography using a HisTrap column (GE Healthcare). C1 was obtained from Complement Technology Inc. (TX, USA).

### Complement-Binding Molecules and Proteins

FH and C4b-binding protein (C4BP) were ordered *via* Complement Technology Inc. (Tyler, TX, USA). FHR5 was purchased at R&D systems (Minneapolis, MN, USA). Eculizumab was obtained *via* Genmab (Utrecht, The Netherlands). Cp40 was kindly provided by John Lambris. CRIg was kindly provided by Genentech (South San Francisco, CA, USA). OmCI was produced in HEK293E cells and purified as described previously (20). Efb-C and Efb-C mutant (Efb-C-R131E/N138E) were prepared as previously described (21, 22), as well as Ecb, Ecb mutant (Ecb-N63E/R75E/N82E) (23), and SSL7 (24).

### Human Monoclonal Antibodies

Monoclonal human anti-DNP-IgG1 was produced recombinantly in human Expi293F cells (Life Technologies). Therefore, the variable region of the heavy chain (>VH7007-DNP-G2a2: DVRLQESGPGLVKPSQSLSLTCSVTGYSITNSYYWNWIRQFPGNKLEWMVYIGYDGSNNYNPSLKNRISITRDTSKNQFFLKLNSVTTEDTATYYCARATYYGNYRGFAYWGQGTLVTVSA) and light chain (>VL7007-DNP-G2a2: DIRMTQTTSSLSASLGDRVTISCRASQDISNYLNWYQQKPDGTVKLLIYYTSRLHSGVPSRFSGSGSGTDYSLTISNLEQEDIATYFCQQGNTLPWTFGGGTKLEIK) (25) were cloned in the pFUSE-CHIg-hG1 and pFUSE2-CLIg-hk vector, respectively, according to the manufacturer’s description (Invivogen). A KOZAK sequence and the HAVT20 signal peptide (MACPGFLWALVISTCLEFSMA) were included upstream each variable region. Human codon optimized sequences were ordered as gBlocks (Integrated DNA Technologies) for Gibson assembly (Bioke). TOP10F′ *E. coli* were used for propagation of the plasmids. After sequence, verification plasmids were isolated using NucleoBond Xtra Midi plasmid DNA purification (Macherey-Nagel). Transfection of EXPI293F cells was performed using ExpiFectamine 293 reagent according to the manufacturer’s description (Life Technologies). 1 µg DNA/ml cells was used in a 3:2 (hk:hG1) ratio. Cell supernatant was collected after 4 days of transfection and antibodies were isolated using a HiTrap protein A column (GE Healthcare).

### U937 Cell Lines

U937 human monocyte cells and 293 T human embryonic kidney cells were obtained from American Type Culture Collection and grown (37°C, 5% CO_2_) in RPMI (Lonza) supplemented with penicillin and streptomycin (Gibco) and 10% FCS (Gibco). For stable expression of human C3aR in U937 cells, a lentiviral expression system was used. The human C3aR cDNA was cloned in a dual promoter lentiviral vector, derived from no. 2025.pCCLsin.PPT.pA.CTE.4x-scrT.eGFP.mCMV.hPGK.NGFR.pre (kindly provided by Dr. Luigi Naldini, San Raffaele Scientific Institute, Milan, Italy) as previously described (26). This altered lentiviral vector (BIC-PGK-Zeo-T2a-mAmetrine; EF1A) uses the human EF1A promoter to facilitate potent expression in immune cells and expresses the fluorescent protein mAmetrine and selection marker ZeoR. Virus was produced in 24-well plates using standard lentiviral production protocols and the third-generation packaging vectors pMD2G-VSVg, pRSV-REV, and pMDL/RRE. Briefly, 0.25 µg lentiviral vector and 0.25 µg packaging vectors were co-transfected in 293 T cells by using 1.5 µl Mirus LT1 tranfection reagent (Sopachem, Ochten, The Netherlands). After 72 h, 100 µl viral supernatant adjusted to 8 mg/ml polybrene was used to infect ~50,000 U937 cells by spin infection at 1,000 g for 2 h at 33°C. U937-C5aR cells were a generous gift from Eric Prossnitz (University of New Mexico, Albuquerque, NM, USA).

### C3 and C5 Conversion in AP Model

Streptavidin-coated magnetic beads (Dynabeads M-270 Streptavidin, Invitrogen) were washed once in VBS-T/Mg [Veronal Buffered Saline pH 7.4, 2.5 mM MgCl_2_, 0.05% (v/v) Tween]. To prepare fully loaded C3b-beads, beads (4 µl/sample) were resuspended in 0.4 ml VBS-T/Mg per sample with C3b-PEG11-biotin (1 µg/ml) and incubated for 1 h at 4°C on roller. To load beads with different amounts of C3b, five different amounts of beads per sample (4, 8, 16, 32, or 64 µl beads) were incubated in 0.4 ml VBS-T/Mg with 0.6 µg/ml C3b-PEG11-biotin. After C3b-labeling, beads were washed three times and incubated in 100 µl VBS-T/Mg per sample with FB (50 µg/ml) for 30 min at room temperature on roller. After FB incubation, beads were washed three times and incubated in 100 µl VBS-T/Mg per sample with FD (5 µg/ml) and either C3 (20 µg/ml) or C5 (20 µg/ml) and with or without inhibitor at the desired concentration (1 µM or threefold dilution starting from 1 µM) for 1 h at 37°C on shaker. After incubation, supernatant of each sample was collected and kept at −20°C until measurement in calcium mobilization assay.

### C3 and C5 Conversion in CP Model

Streptavidin-coated magnetic beads (Dynabeads M-270 Streptavidin, Invitrogen) were washed once in PBS-TH [Phosphate Buffered Saline pH 7.4, 0.05% (v/v) Tween, 0.5% HSA]. Beads (4 µl/sample) were resuspended in 0.4 ml PBS-TH per sample with 1 µg/ml biotinylated 2,4-dinitrophenol [DNP-PEG2-GSGSGSGK(Biotin)-NH2; 1,186 Da; obtained from Pepscan Therapeutics B.V., The Netherlands] and incubated for 30 min at 4°C on roller. Beads were washed once in PBS-TH and incubated in 0.2 ml PBS-TH per sample with 10 nM human monoclonal anti-DNP-IgG1 for 30 min at 4°C on roller.

After one wash in PBS-TH, beads were incubated in 0.1 ml VBS^++^-TH [Veronal Buffered Saline pH 7.4, 0.25 mM MgCl_2_, 0.5 mM CaCl_2_, 0.05% (v/v) Tween, 0.5% HSA] per sample with 0.8 µg/ml C1 for 30 min at 37°C, shaking. Beads were washed three times in VBS^++^-TH and incubated in 0.1 ml VBS^++^-TH per sample with 10 µg/ml C4 for 30 min at 37°C, shaking. After three washes in VBS^++^-TH, beads were incubated in 0.1 ml VBS^++^-TH per sample with 10 µg/ml C2, 10 µg/ml C3, and 0.5 µg/ml C5 with or without 1 µM inhibitor for 5 min at 37°C on shaker. After incubation, the supernatant of each sample was collected and kept at −20°C until measurement in calcium mobilization assay.

In some CP experiments, the amount of deposited C3b was influenced by adding lower concentrations of C3 (threefold decrease starting from 10 µg/ml). In one condition, C3 and C5 conversion were separated by incubating beads first in 0.1 ml VBS^++^-TH per sample with 10 µg/ml C2 and 10 µg/ml C3 for 5 min at 37°C and subsequently, after washing, in 0.1 ml VBS^++^-TH with 10 µg/ml C2 and 0.5 µg/ml C5 for 5 min at 37°C. Supernatant of both C2 + C3 and C2 + C5 incubation were here collected and used for calcium mobilization in U937-C3aR and U937-C5aR cells, respectively. Controls were carried out with 10 µg/ml C3b_H2O_ or 10 µg/ml C3_MA_.

### Calcium Mobilization Assay With U937 Cells

U937-C3aR and U937-C5aR cells were washed in RPMI/0.05% HSA and diluted to 5 × 10^6^ cells/ml. Cells were incubated with 0.5 µM Fluo-3-AM (Invitrogen) on roller in dark at room temperature for 20 min, washed and resuspended in RPMI/1% HSA to a final concentration of 1 × 10^6^ cells/ml. For calcium mobilization measurements, the labeled cells were stimulated with sample supernatant (ratio cells to supernatant is 9:1) while cell fluorescence is measured by flow cytometry (BD FACSVerse) from 10 s before until 40 s after addition of the sample. The absolute calcium mobilization was calculated by subtracting the cell mean fluorescence intensity (MFI) before cell activation (*t* = 5–15 s) from the MFI after stimulation (*t* = 30–50 s) using FlowJo software. Standard curves were obtained using 10-fold dilutions starting from 1 µM of C3a (Complement Technology Inc., TX, USA) or C5a (Bachem, Switzerland) as stimuli for the cells. As negative controls the calcium mobilization in U937-C3aR and U937-C5aR cells induced by 0.1 µM C3 or C5 was measured.

### C3b Binding to Beads

To determine the level of C3b-biotin bound to streptavidin-coated beads in the AP model or the level of actively deposited C3b in the CP model, beads were washed three times in PBS-T (AP) or PBS-TH (CP) after the C3b-biotin or C3 incubation and incubated (4 µl beads/sample) in 100 µl PBS-TH per sample with 1:100 FITC-conjugated goat-anti-human C3 (Protos) for 30 min at 4°C. Subsequently, beads were washed three times in PBS-T (AP) or PBS-TH (CP) and C3b binding was analyzed by flow cytometry (BD FACSVerse).

### Statistical Analysis

Statistical analysis was performed with GraphPad Prism 6 software. All calcium flux data are presented as mean ± SD from three independent experiments. C3b binding data are presented as geometric mean ± SD from three independent experiments.

## Results

### Functional Analysis of C3 Conversion *via* Purified AP Convertases

**Figure 2:**
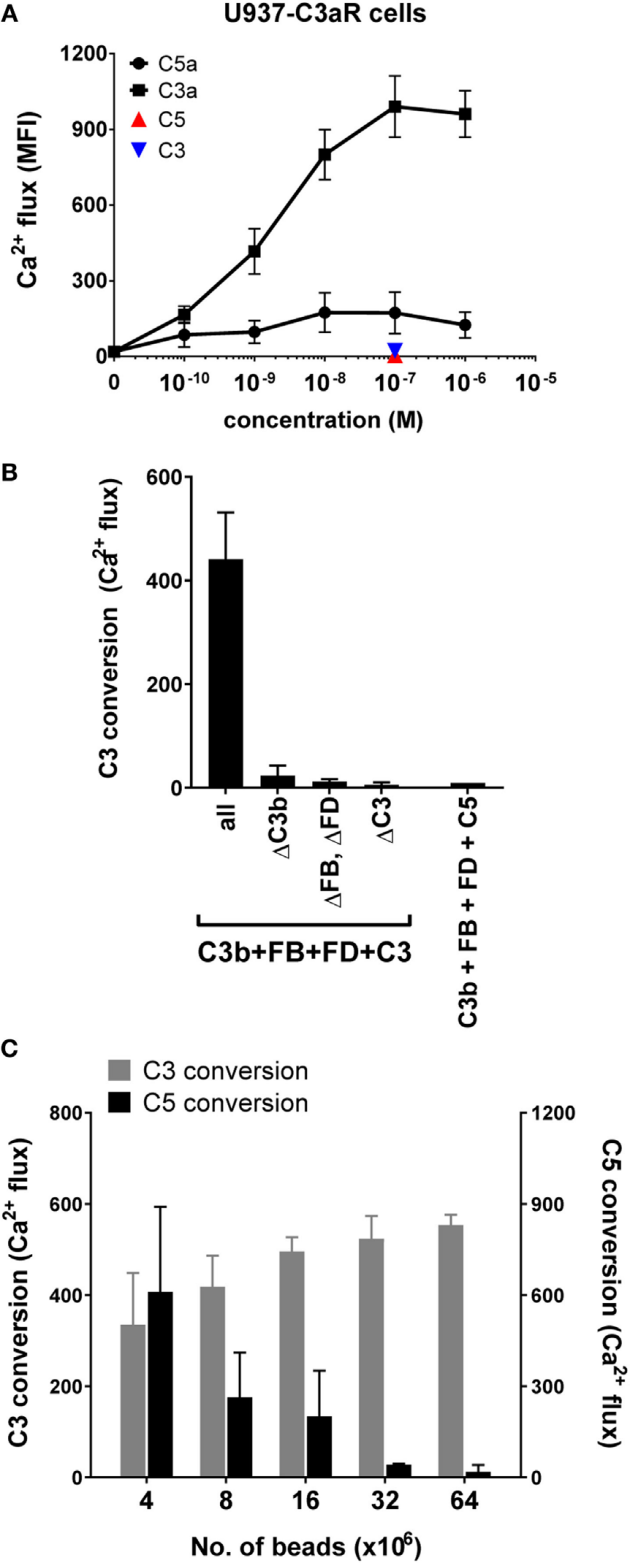
Development of an alternative pathway (AP) C3 convertase model. **(A)** C3a specifically induces calcium mobilization in U937-C3aR cells, while C3 and C5 do not. **(B)** C3 conversion by AP convertases on beads was analyzed in a calcium mobilization assay with U937-C3aR cells. C3a could only be detected in the sample supernatant in the presence of all AP components. C5 conversion in the AP model did not induce calcium flux in the U937-C3aR cells. **(C)** AP C3 and C5 conversion were performed on beads coated with different densities of C3b and analyzed by calcium mobilization assay with U937-C3aR and -C5aR cells, respectively. A high density of C3b molecules on the target surface enhances C5 but not C3 conversion. **(A–C)** Data of three independent experiments, presented as mean ± SD.

Previously, we described the development of a model system to study C5 convertases of the AP using purified components (7). The AP C5 convertase is formed when C3 convertases (C3bBb) cleave C3 into nascent C3b that covalently binds to target surfaces *via* the thioester (structural model in Figure 1C) (27). At a critical density of C3b molecules, multimeric C3b_n_ complexes arise that have a high affinity for C5. These C3b_n_ complexes, together with FB and FD, generate C5 convertases that bind and cleave C5 (Figure 1D). We recently set out to mimic surface-bound high C3b-density using purified proteins. To establish this, we first labeled C3b with biotin *via* the thioester by activating plasma-purified C3 into C3b in the presence of a biotinylation agent that reacts with the cysteine residue of the C3b thioester (28). These biotinylated C3b molecules were subsequently loaded onto small magnetic streptavidin (SA) beads (2.8 µm diameter) and incubated with FB and FD to form surface-bound convertases. C5 conversion was examined by quantifying the release of C5a using a flow cytometry-based calcium mobilization assay (29, 30). In short, U937 cells transfected with the C5a receptor (U937-C5aR) were exposed to sample supernatants containing C5a. Binding of C5a to the C5a receptor mediates intracellular calcium release, which is detected by a fluorescent indicator. To get more insights into convertase formation and inhibition, we here extended this model to also study C3 conversion by purified AP convertases. First, we transfected U937 cells with the C3a receptor (U937-C3aR) and showed that purified C3a, but not C3, successfully triggers the mobilization of intracellular calcium in U937-C3aR cells (Figure 2A). Low-level activation by purified C5a is likely due to low levels of endogenous C5aR expression in these cells (Figure 2A). Second, C3b-coated beads were incubated with FB, FD and C3 and release of C3a was determined in supernatants *via* calcium mobilization in U937-C3aR (Figure 2B). C3a-dependent calcium flux specifically required all convertase components. In our previous study, we determined that high C3b-density is essential to effectively convert C5 (7). By maintaining a constant concentration of C3b in each sample while increasing the number of beads, we artificially lowered the local concentration of C3b on the surface (Figure S1 in Supplementary Material). Here, we find that while lowering C3b surface density reduces C5 conversion by AP convertases, it does not lead to less C3 conversion (Figure 2C). These results demonstrate key differences in the conditions required for C3 and C5 conversion on a surface in the AP.

### Select C3b-Binding Molecules Inhibit AP C5 but Not C3 Conversion

**Figure 3:**
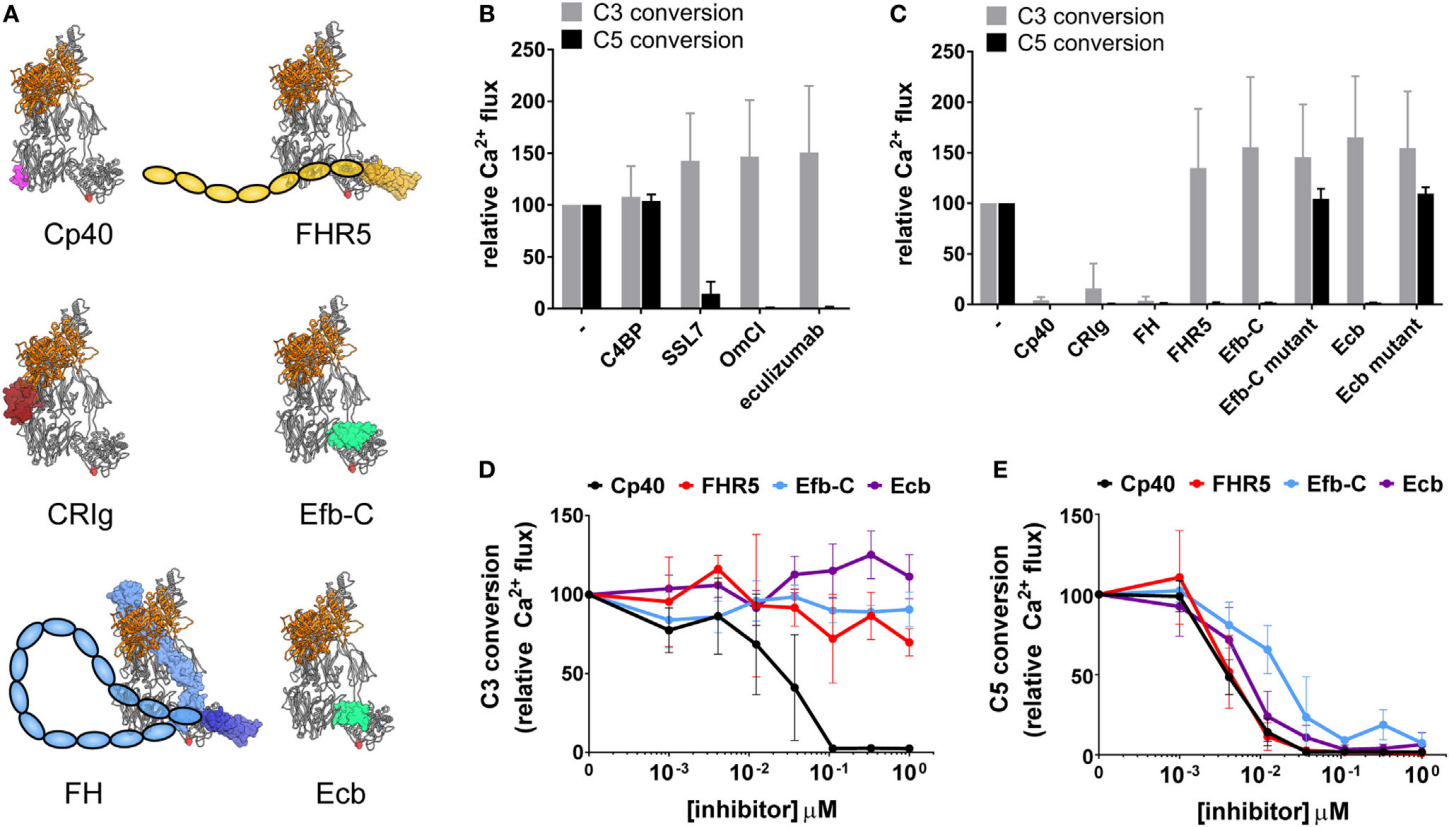
C3b-binding molecules FHR5 and Efb-C/Ecb selectively inhibit C5 conversion by alternative pathway (AP) convertases. **(A)** Structural models of the C3bBb convertase with C3b-binding molecules. The structure of C3bBb [from the C3bBb-SCIN dimer structure, PDB 2WIN (55)] is identical to the convertase shown in Figures 1C,D with C3b (gray) and Bb (orange) shown as ribbons, and in the same orientation, with the C3b–C3b dimerization site (as shown in Figure 1C, right) on the left side of the convertase. C3b-binding molecules are shown as molecular surfaces; Cp40 (magenta) is based on the C3c–compstatin complex structure [PDB 2QKI (41)], CRIg (dark red) is based on the C3b–CRIg complex structure [PDB 2ICF (31)], FH is based on the C3b–FH (1–4) structure [light blue, PDB 2WII (43)] and the C3d–FH (19–20) structure [dark blue, PDB 2XQW (44)], FHR5 (yellow) is modeled from the C3d–FH (19–20) structure [PDB 2XQW (44)], Efb-C (spring green) is based on the C3d–Efb-C structure [PDB 2GOX (21)], and Ecb (spring green) is based on the C3d–Ehp structure [PDB 2NOJ (23)]. For FH and FHR5 only some domains are structurally resolved. Additional domains are represented by colored ovals. **(B–E)** Conversion of C3 and C5 (0.1 µM) in the AP convertase model in the presence of complement-binding molecules measured by calcium mobilization in U937-C3aR and U937-C5aR cells, respectively. Conversion is shown as a percentage relative to the control without inhibitor. Data of three independent experiments, presented as mean ± SD. **(B)** C5-binding molecules SSL7, OmCI, and eculizumab (all 1 µM) inhibit AP C5 but not C3 conversion. C4b-binding protein has no effect in the AP model. **(C)** C3b-binding molecules Cp40, CRIg, and FH prevent both C3 and C5 conversion, while FHR5, Efb-C, and Ecb selectively inhibit C5 conversion. Mutant Efb-C and mutant Ecb are unable to bind C3b and thus do not exhibit inhibition. **(D)** FHR5, Efb-C, and Ecb do not affect C3 conversion, but **(E)** selectively inhibit C5 conversion in a dose-dependent manner. Cp40 was used as positive control, inhibiting both C3 and C5 conversion.

Having established a system to study both C3 and C5 conversion by AP convertases, we could now dissect whether known convertase inhibitory molecules block cleavage of both substrates. We focused on studying C3b- or C5-binding molecules for which the binding sites to C3b/C5 have been determined. Each of these molecules has been extensively characterized in previous studies and is known to influence convertase activity in physiological environments (Table 1). The structures of the C3b-binding molecules in complex with the C3bBb convertase are shown in Figure 3A. The inhibitors include naturally occurring complement inhibitors derived from humans [C3b-binders CRIg (31, 32), FH (33, 34), and FHR5 (35)], bacteria [homologous C3b-binders Efb-C (36) and Ecb (16, 23) and C5-binder SSL7 (37)], and ticks [C5-binder OmCI (20)], or therapeutic inhibitors eculizumab [Soliris, a clinically approved antibody against C5 (38)] and Cp40 [a compstatin analog, strong C3- and C3b-binding molecule (39)]. As expected, we observed that the three C5-binding molecules SSL7, OmCI, and eculizumab specifically interfered with C5 conversion but not C3 conversion (Figure 3B). C4BP (40) was included as a negative control for inhibition, as it should not have an effect on AP C3 or C5 convertases, since these convertases lack C4b (Figure 3B). Next, we analyzed the activity of C3b-binding molecules on C3 versus C5 conversion. While C3b-binding molecules Cp40, CRIg, and FH blocked both C3 and C5 conversion by AP convertases, host regulatory protein FHR5, and staphylococcal immune evasion proteins Efb-C and Ecb specifically blocked C5 conversion, while leaving C3 conversion unaffected (Figure 3C). Mutants of Efb-C/Ecb proteins that cannot bind C3b could not inhibit C5 conversion, confirming that the observed inhibition is mediated through interaction with C3b (Figure 3C) (21, 23). Furthermore, Efb-C, Ecb, and FHR5 all inhibit AP C5 conversion in a concentration-dependent manner (Figure 3E), but do not affect C3 conversion (Figure 3D). As a control, we observed that Cp40, which showed inhibition of both AP C3 and C5 conversion at 1 µM concentration, also inhibits both C3 and C5 conversion in a concentration-dependent manner (Figures 3D,E). Thus, these data suggest that by binding C3b, selective inhibition of AP C5 convertases is possible. AP C5 convertases are similar to AP C3 convertases, but contain accessory C3b molecules (C3b_n_) that enable efficient C5 conversion on surfaces. Since Efb-C, Ecb, and FHR5 only affect C5 (and not C3) conversion, they likely inhibit through affecting accessory C3b molecules on the bead surface. However, the fact that AP C3 and C5 convertases both contain C3b confounds the ability to independently study the role of accessory C3b molecules in C5 conversion.

**Table 1:** Overview of complement inhibitors used in this study.

Inhibitor		Type	Key references
**C3b-binding molecules**			
Cp40	Compstatin (analog Cp40)	Cyclic peptide	(39, 41, 42)
CRIg	Complement receptor of immunoglobulin family	Human complement regulator protein	(31, 32)
FH	Factor H	Human complement regulator protein	(33, 34, 43, 44)
FHR5	Factor H related-protein 5	Human complement regulator protein	(35, 45)
Efb-C	(C-terminal region of) Extracellular fibrinogen binding protein	*Staphylococcus aureus* immune evasion protein	(21, 36, 46)
Ecb	Extracellular complement binding protein (also known as Ehp)	*Staphylococcus aureus* immune evasion protein	(16, 23)
**C5-binding molecules**			
SSL7	Staphylococcal superantigen-like protein 7	*Staphylococcus aureus* immune evasion protein	(37, 47, 48)
OmCI	Ornithodoros moubata complement inhibitory protein	*Ornithodoros moubata* immune evasion protein	(20, 49, 50)
Eculizumab	Also known as Soliris	Humanized monoclonal antibody	(38, 51, 52)
**C4b-binding molecules**			
C4BP	C4b-binding protein	Human complement regulator protein	(40, 53, 54)

### Development of a Purified Classical Pathway C3/C5 Convertase Model

**Figure 4:**
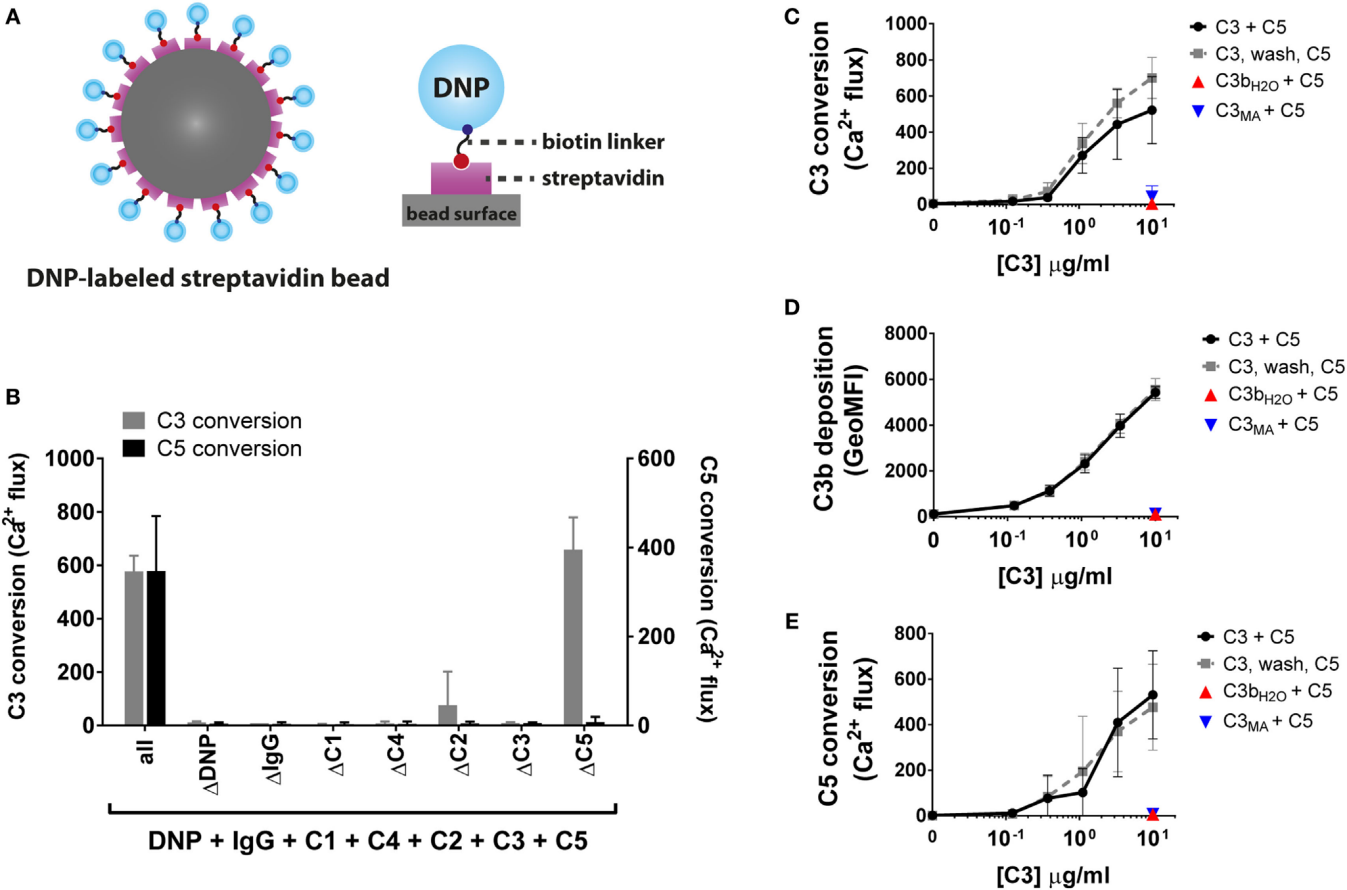
Development of a classical pathway C3 and C5 convertase model. **(A)** In the CP convertase model, streptavidin beads are labeled with biotinylated DNP which serves as a model antigen. Addition of IgG1 recognizing DNP and complement proteins results in formation of C4b2a and C4b2a(C3b)_n_ convertases on the bead surface, which convert C3 and C5. **(B)** Only in the presence of all CP components (antigen, IgG, and CP proteins) C3 and C5 are converted, as measured by calcium mobilization in U937-C3aR and U937-C5aR cells, respectively. **(C)** C3 conversion *via* CP convertases results in release of C3a in the supernatant (as shown by calcium mobilization) and **(D)** C3b deposition on the bead surface (as shown by flow cytometry). Identical amounts of the non-reactive C3 variants C3b_H2O_ and C3_MA_ do not bind to the bead surface. **(E)** C5 convertase activity increases with the level of deposited C3b molecules on the beads surface, but is not affected by C3b_H2O_ or C3_MA_ in solution. Uncoupling C3 and C5 conversion by introduction of an extra washing step does not alter C5 conversion, indicating that CP C5 conversion only depends on deposited C3b molecules around existing C3 convertases. **(B–E)** Data of three independent experiments, presented as mean ± SD.

To more closely investigate convertase inhibitory mechanisms of complement inhibitors, we next developed a model to study C3/C5 conversion *via* purified CP convertases. In this pathway, modulation of accessory C3b molecules can be better analyzed since the CP C3 convertase (C4b2a) does not contain C3b. We have used a model that fully recapitulates the CP activation pathway using purified complement components. Streptavidin beads were labeled with biotinylated DNP antigen (Figure 4A) and sequentially incubated with recombinant human IgG1 recognizing DNP, C1, C4, C2 and substrates C3 and C5. Similar to the AP, we detected the release of C3a and C5a in the sample supernatant *via* calcium mobilization in U937-C3aR and U937-C5aR cells, respectively. The lack of calcium mobilization by the sample supernatants in the absence of DNP, IgG or the individual complement proteins demonstrates the necessity of functional CP convertases on the bead surface for C3/C5 cleavage (Figure 4B). Our results also showed that there was no cross-reactivity between mismatched ligands and receptors. The absence of calcium flux in U937-C5aR cells by samples lacking C5 (and thus C5a) showed that C3a generated in these samples does not interfere with C5a detection. As calcium mobilization levels in U937-C3aR cells were not affected by the presence of C5, interference of C5a with C3a-specific detection could be excluded, as well. Unlike our AP C5 convertase model where we artificially coupled C3b to the bead surface, deposition of C3b in the CP could only be established by natural C3 conversion *via* C4b2a. By adding different concentrations of C3 to beads with naturally formed C4b2a convertases, we influenced the level of C3 conversion and thus C3b deposition on the bead surface (Figures 4C,D). Indeed, CP C5 conversion was highly dependent on the level of deposited C3b (Figure 4E). As a control, we showed that C5 conversion specifically depends on covalently deposited C3b since addition of non-reactive C3 variants [C3b_H2O_ in which the thioester had already reacted with water or methylamine-treated C3 (C3_MA_) (7, 56)] did not induce the convertase to cleave C5 (Figures 4C–E). In addition, introducing a washing step in between C3b deposition and C5 conversion confirmed that only deposited C3b and not C3 or the active process of C3 cleavage is required for C5 conversion (Figure 4E). These results demonstrate the specificity of our CP convertase models in measuring the activity of CP C3 and C5 conversion.

### C3b-Binding Molecules Inhibit Classical Pathway C5 Conversion by Modulating Accessory C3b

**Figure 5:**
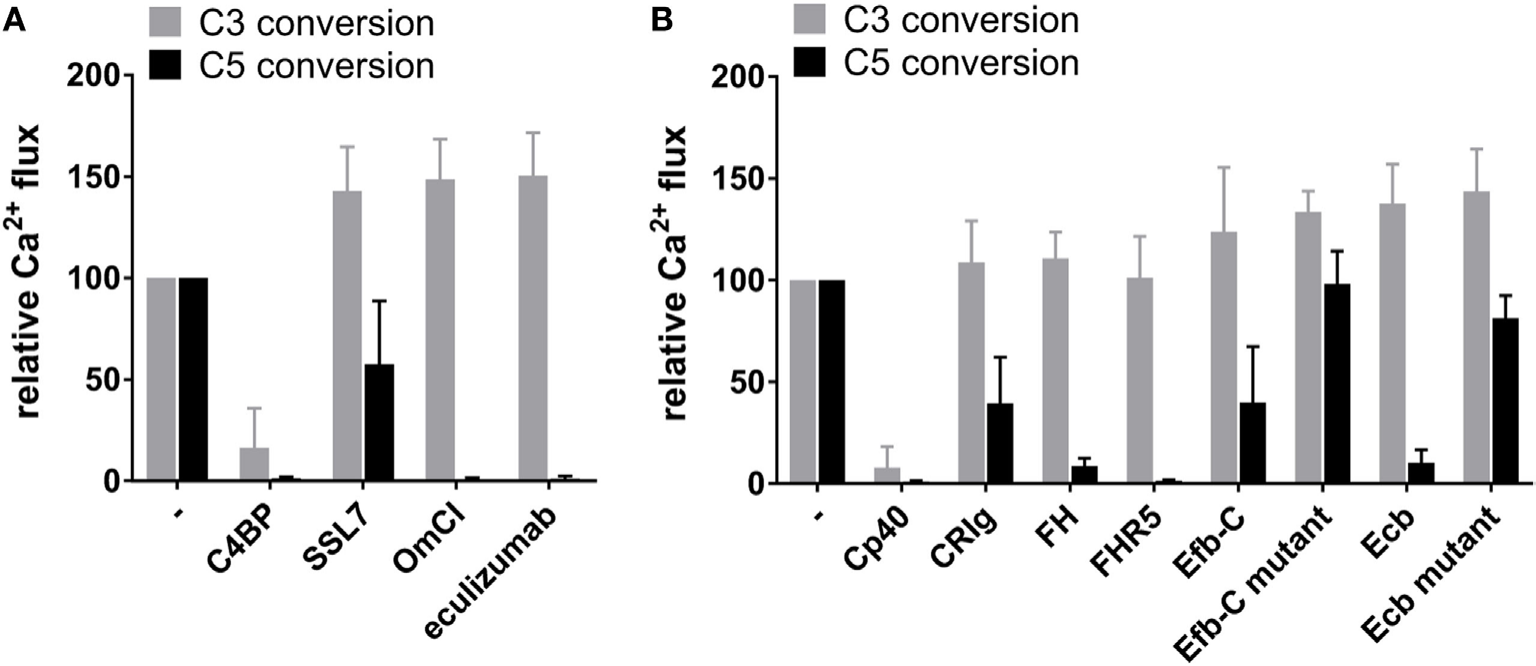
The effect of inhibitors on classical pathway C3 and C5 conversion. Conversion of C3 (50 nM) or C5 (2.5 nM) in the CP model in the absence or presence of 1 µM complement-binding molecule measured by calcium mobilization in U937-C3aR and U937-C5aR cells, respectively. Conversion is shown as a percentage relative to the control without inhibitor. **(A)** C5-binding molecules OmCI and eculizumab inhibit CP C5 but not C3 conversion. SSL7 inhibits C5 conversion, as well, but less efficiently. C4b-binding protein (C4BP) inhibits both CP C3 and C5 convertases. **(B)** None of the C3b-binding molecules, except for Cp40, affect CP C3 conversion, whereas all inhibit C5 conversion. Mutant Efb-C and mutant Ecb are unable to bind C3b and thus do not exhibit inhibition. **(A,B)** Data of three independent experiments, presented as mean ± SD.

Next, we examined the effect of the above-tested C3b- and C5-binding molecules in the CP convertase model. Beads with actively formed C4b2a convertases were incubated with C2, C3, and C5 in the presence of complement binding molecules at a concentration of 1 µM and the C3a/C5a generated in the sample supernatant was measured. The known CP convertase inhibitor C4BP effectively inhibited both CP C3 and C5 conversion, confirming the validity of our model (Figure 5A). Furthermore, C5-binding molecules OmCI and eculizumab potently inhibited CP C5 conversion but left C3 conversion unaffected (Figure 5A). SSL7 also inhibits CP C5 conversion, but to a lesser extent, which could arise from differences in the models or in the C5-binding sites involved in convertase recognition. Since C3b is not part of the CP C3 convertase, we hypothesized that C3b-binding molecules would not influence C3 conversion in the CP model. Accordingly, most C3b-binding molecules did not affect C3 conversion, with the exception of Cp40, which showed potent inhibition (Figure 5B). The lack of C3 conversion in the presence of Cp40 can be explained by its strong affinity for uncleaved C3 causing steric hindrance during C3 recognition by the convertase (57). Efb-C and Ecb, which can also bind C3, do not inhibit convertase activity in that manner, as further evidenced by lack of inhibition of AP C3 conversion. Interestingly, we found that all C3b-binding molecules can prevent C5 conversion in the CP model (Figure 5B). This establishes an important role for accessory C3b in the formation and activity of CP C5 convertases. Moreover, the fact that FHR5, Efb-C, and Ecb exhibited similar effects on C5 conversion by the AP and CP convertases, indicates a similar role for accessory C3b in C5 conversion in both pathways. The CP model provides more detail about their inhibitory mechanism by showing that they can act specifically *via* accessory C3b molecules. Similarly, the CP inhibition data show that CRIg and FH can inhibit C5 conversion specifically *via* accessory C3b. Overall, our data suggest that all C3b-binding molecules tested can inhibit C5 conversion (in both AP and CP) through interaction with accessory C3b molecules, but only some can inhibit the core convertase enzyme (C3bBb) itself.

## Discussion

Since C3 and C5 convertase enzymes play such a vital role in propagating the cascade but also driving unwanted complement effector functions, it is essential to better understand mechanisms of convertase activation and inhibition. Since C5 convertases are largely constrained to cell surfaces, it has been difficult to study these enzymes with highly purified complement components. Here, we developed bead-based models to functionally characterize both C3 and C5 convertases on a surface using purified components and in the absence of confounding factors from serum. These models serve several important purposes: (1) to understand the molecular biology of convertases, (2) to characterize the mode of action of known complement inhibitors, (3) to characterize the role of disease-associated deficiencies and mutations of complement proteins, and (4) to screen for novel and specific convertase inhibitors.

In the past, several models have been developed to study convertase activation and inhibition. One of the most common models employs erythrocytes to serve as a platform for complement activation, using either serum or stepwise addition of purified complement components. Other studies have employed non-cellular surfaces, including SPR chips, to examine stepwise assembly and dissociation of convertases. While each of these models differ in many aspects, each has inherent advantages and disadvantages in addressing various aspects of complement function, and no single model can capture all molecular and physiological details of convertases or inhibition thereof. Our models offer the ability to (1) quantitatively compare activity and inhibition of AP and CP C3 and C5 convertases independently and (2) enable controlled formation and distribution of convertases in a highly purified environment in the absence of complement regulators found in serum and on cells. In our model, we chose to quantify C3 and C5 cleavage through measurement of chemoattractants C3a and C5a, molecules that are released into solution and can be selectively and sensitively detected in a functional cell-based calcium mobilization assay using flow cytometry. Alternatively, C3a and C5a can be quantified by ELISA, however, this is not a direct functional readout, and one should exercise caution in selecting antibodies with high specificity for each chemoattractant molecule (58). Measurement of C3a *via* calcium responses allows a more accurate quantification of C3 convertase activity than antibody detection of deposited C3b molecules. During C3 cleavage, the thioester of newly formed C3b molecules becomes exposed and can react with molecules on the cell surface. Rapid amplification results in dense clusters of C3b, which may deposit on top of each other, making accurate quantification difficult (59). Furthermore, many newly formed C3b molecules never attach to the surface (60). Therefore, immunodetection of deposited C3b is not the best measure of C3 conversion. In addition, since C3a and C5a are hallmarks of complement-mediated inflammation, detection of these chemoattractants is a critical readout when screening for convertase inhibitors as potential therapeutic molecules and disease-associated mutants of complement factors *in vitro*. It is important to note that in more complex environments (i.e., serum or *in vivo*), measurement of functionally active C3a/C5a is challenging due to proteolytic cleavage and scavenging by receptors. In addition, our bead-based models enable additional readouts, including quantification of surface complement deposition and breakdown of complement opsonins through cofactor activity of inhibitory molecules (Figure S2 in Supplementary Material).

**Figure 6:**
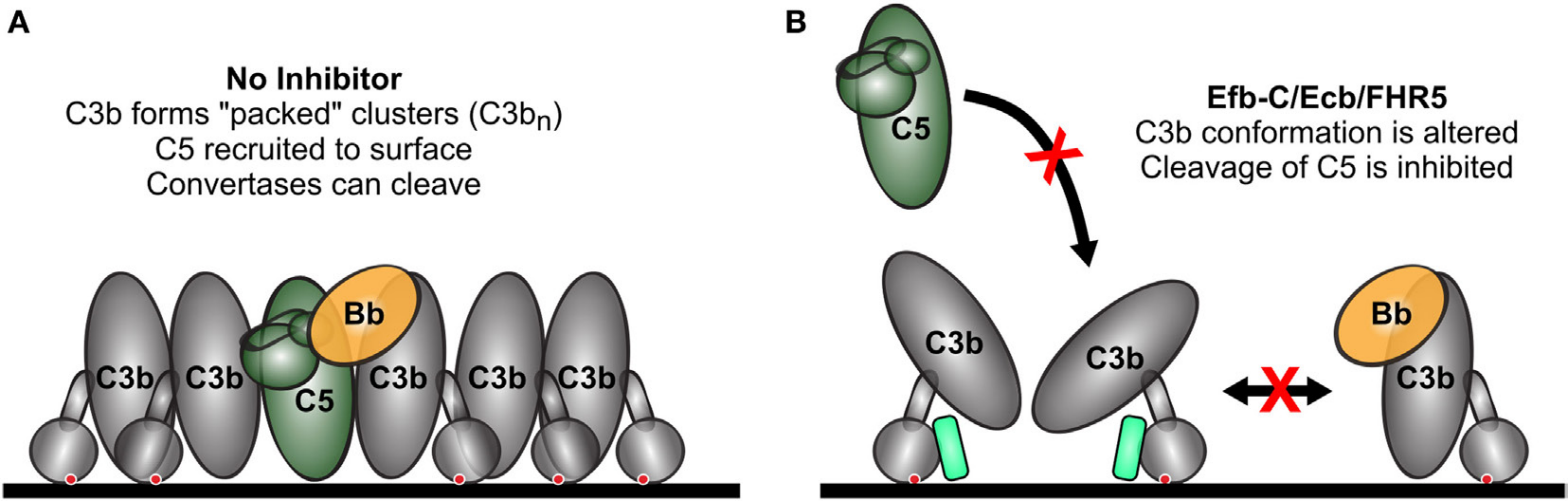
Model for selective inhibition of C5 conversion. **(A)** Under normal conditions, high levels of accessory C3b (C3b_n_) deposition on a cell surface around existing C3 convertases enables binding and conversion of C5. All C3b molecules are tightly packed and aligned in the same vertical orientation, both of which are necessary for efficient C5 conversion. **(B)** Thioester domain (TED)-binding molecules (i.e., Efb-C and Ecb) act as a wedge to separate TED from the rest of C3b, which alters its surface conformation and prevents efficient C5 conversion. Colors of molecules correspond to Figures 1C,D and 3A, with Efb-C/Ecb shown in spring green. The red crosses indicate inhibition of C5 and C3bBb binding to surface-bound accessory C3b molecules (C3b_n_).

The models presented in this work may assist in obtaining better insights into the structural organization of convertase enzymes. While significant progress has been made in understanding the structural organization of C3 convertases and C3 cleavage (Figure 1C) (55), the molecular details of C5 convertase formation remain poorly understood. Molecular models of C5 convertase activation have been proposed (Figure 1D) (47, 49, 61) but the exact organization of this complex remains unknown. It is known that C5 convertases form when C3 convertases (C4b2a and C3bBb) deposit high densities of C3b molecules on the target surface (9). The non-catalytic subunits of C3 convertases (C4b or C3b) are thought to associate with extra C3b molecules and form multimeric C4b-C3b_n_ or C3b-C3b_n_ complexes that have an increased affinity for C5 (62, 63). In this study, we verified the requirement of high C3b densities for C5 conversion. In line with previous data, we also find that C3b density affects C5, but not C3 conversion by AP convertases (7). Interestingly, our data for Efb-C/Ecb and FHR5 also suggest that the orientation of C3b molecules on the surface is particularly important for conversion of C5, but not C3. We previously demonstrated that the (natural) surface attachment of C3b molecules *via* the thioester is crucial for efficient conversion of C5 (7). Here, we found that three molecules that interact with the C3b thioester domain (TED) (Efb-C, Ecb, and FHR5) selectively inhibit AP and CP C5 convertases, while leaving C3 conversion unaffected. Among examined C3b-binding molecules, this selective inhibition of C5 conversion in the AP was specific for molecules interacting with the TED of C3b. Several crystallographic structures of C3b revealed interdomain interactions between TED and the MG1 domain, which facilitate the prototypical “upright” conformation of C3b attached to surfaces *via* its thioester (31, 43, 55, 64–69). However, recent electron microscopy data reveal conformational flexibility of C3b under different conditions, and in particular, TED can exhibit markedly different positions (70–74). Hydrogen-deuterium exchange experiments demonstrated a conformational change in C3b upon Efb-C binding to TED, suggesting that Efb-C acts as a wedge to disrupt the TED–MG1 interaction and affects the orientation of C3b on the surface (71). Although the exact binding interface of C3b and FHR5 is unknown, it does interact with TED (75), and therefore it is possible that FHR5 acts through a similar mechanism. It is unclear whether FHR5 also interacts with other regions of C3b. Binding of C3b by FHR5 is different from that by FH, because FHR5 lacks domains homologous to the FH N-terminal C3b binding and complement regulatory domains. The results reported here confirm the previously reported lack of solid phase C3 convertase inhibition by FHR5 (45, 76), although inhibition of fluid phase C3 convertase was described (45). Why the C3b orientation is critical for C5 conversion but not C3 conversion remains to be determined. Potentially it supports the recently proposed model in which C5 needs to be “sandwiched” in between the C3 convertase and the accessory C3b molecule in order to be primed for convertase cleavage (49). One could envision that such “sandwiching” is affected by differently oriented C3b’s. Alternatively, C3b orientation may determine how closely C3b molecules can pack together. If tightly packed and aligned accessory C3b molecules are required for efficient C5 conversion, altered orientation may result in decreased conversion of C5 (Figure 6). The fact that Efb/Ecb and FHR5 also inhibit C5 conversion in the CP suggests that the accessory C3b molecules have a similar function in the activation of both the CP and AP C5 convertases.

Functional analyses of well-defined complement inhibitors also reveal other important binding interfaces of C3/C5 convertases. Our model demonstrates that the C3b–C3b dimerization site (Figure 1C, right) is important for activity of all C3b-containing convertases, including AP C3 and C5 convertases, as well as the CP C5 convertase. The C3b-binding molecules Cp40 and CRIg, which bind at this interface (Figure 3A), universally inhibit activity of C3b-containing convertases. These molecules likely interfere with convertase-substrate binding (as in Figures 1C,D) and/or surface-bound accessory C3b molecules (as in Figure 1D). Unlike CRIg, Cp40 inhibits all convertases, including the CP C3 convertase, which lacks C3b. Since Cp40 can bind to both C3b and uncleaved C3, it could inhibit CP C3 conversion by binding to the substrate (C3) and preventing recognition by the CP C3 convertase (57, 77). Substrate C3 binding can also explain the difference in inhibition of AP C3 and C5 conversion by Cp40 (Figures 3D,E). In the AP C3 conversion assay, Cp40 can bind to both C3 and C3b, and therefore the concentration required to block C3 conversion is higher than for in the AP C5 conversion assay, where C3 is not present. Next to CRIg, we also found that FH inhibits CP C5 convertases. Although FH is known as an inhibitor of AP C3/C5 convertases because it dissociates Bb from C3b, the mechanism of FH-mediated inhibition of CP C5 conversion is not known. Since FH is a large molecule with several distant binding sites on C3b (43, 44, 78) it likely interferes with binding C5 (79). Thus, our data for FH demonstrate that not only the C3b–C3b dimerization site, but also other sites on C3b, are important for its interaction with C5. Overall, C3b-binding molecules illustrate several key properties of convertase assembly and inhibition. While much of the data here are in line with previous inhibitor studies (Table 1), more extensive and complementary studies are required to fully understand the physiological modes of inhibition of these molecules.

Finally, the tools developed in this study can be used for identification of effective therapeutic convertase inhibitors. The ability to examine each convertase separately affords the opportunity to identify selective convertase inhibitors. The complement therapeutics landscape is rapidly expanding, as new roles for complement in disease continue to be uncovered. It is clear that not all complement-mediated diseases are created equal, and it is necessary to design therapeutics that target different points in the complement cascade. For example, diseases mediated primarily by C5a or MAC may benefit from selective inhibition of C5 conversion. Blocking the complement cascade upstream of the terminal pathway (i.e., inhibition of C3 cleavage) may unnecessarily increase patient susceptibility to infections by effectively inhibiting all complement effector functions. Our work now demonstrates the characterization of inhibitors that selectively inhibit C5 conversion, which may prove useful in treatment of MAC-mediated and inflammatory disorders of the complement system. Thus, these models provide a platform for the identification of tailored next-generation complement therapeutics.

## Author Contributions

SZ, EB, and SR designed and developed convertase models. SZ, EB, SR, and RG designed the study and experiments. SZ, SM, MR, and RG performed the experiments and analyzed data. CH developed cell lines. CH and PA cloned and produced monoclonal antibodies. SZ, SM, SR, and RG wrote the manuscript. SZ, MJ, SR, and RG contributed to critical analysis and discussion of the results. All authors read and reviewed the manuscript.

## Conflict of Interest Statement

The authors declare that the research was conducted in the absence of any commercial or financial relationships that could be construed as a potential conflict of interest.
