# Supplementary figures and images for: Functional Characterization of Alternative and Classical Pathway C3/C5 Convertase Activity and Inhibition Using Purified Models
Source: Front Immunol. 2018 Jul 23;9:1691. doi: 10.3389/fimmu.2018.01691 (PMC6064732; doi:10.3389/fimmu.2018.01691)

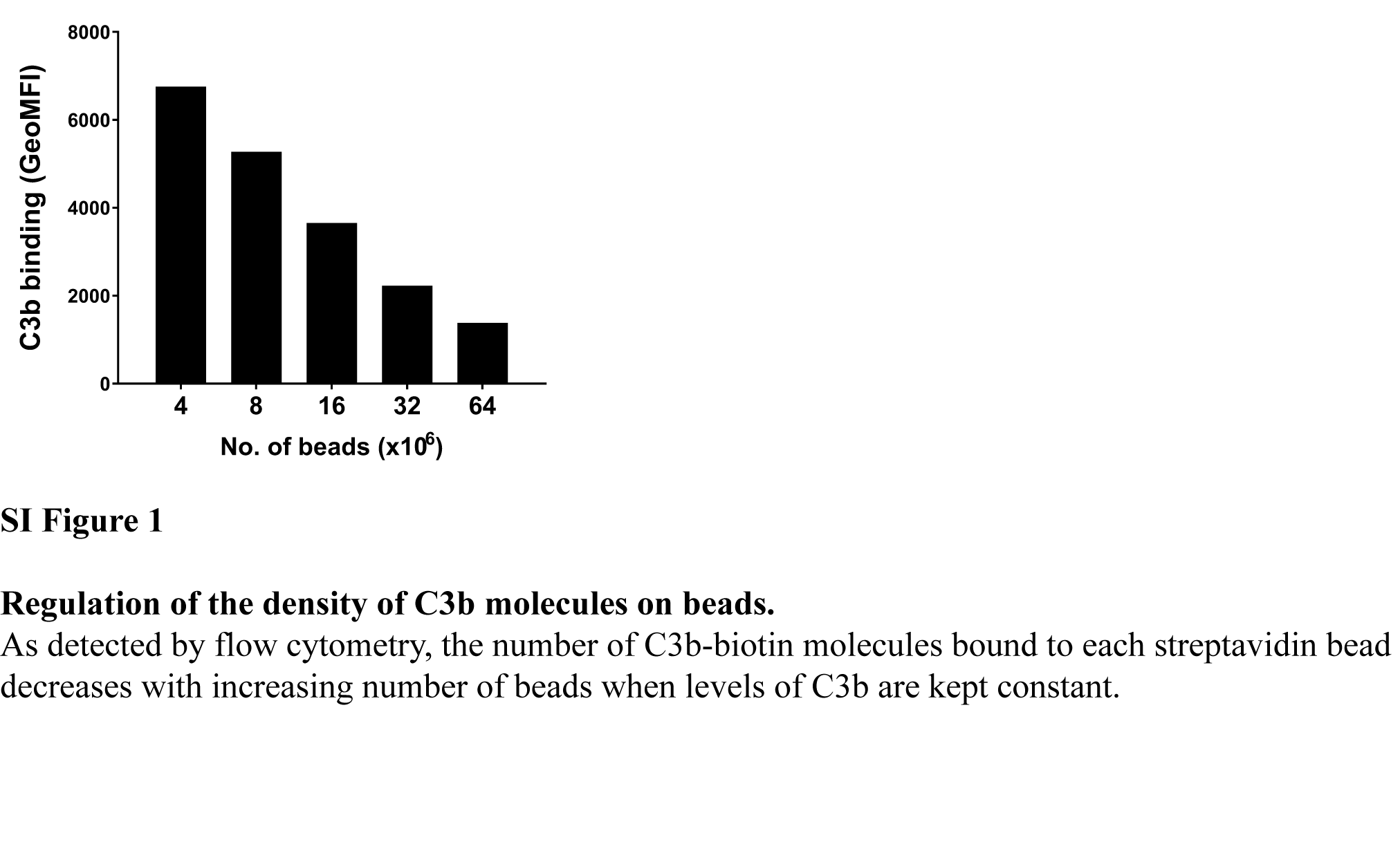

Supplement: Supplementary file 1 [file image_1.tif]

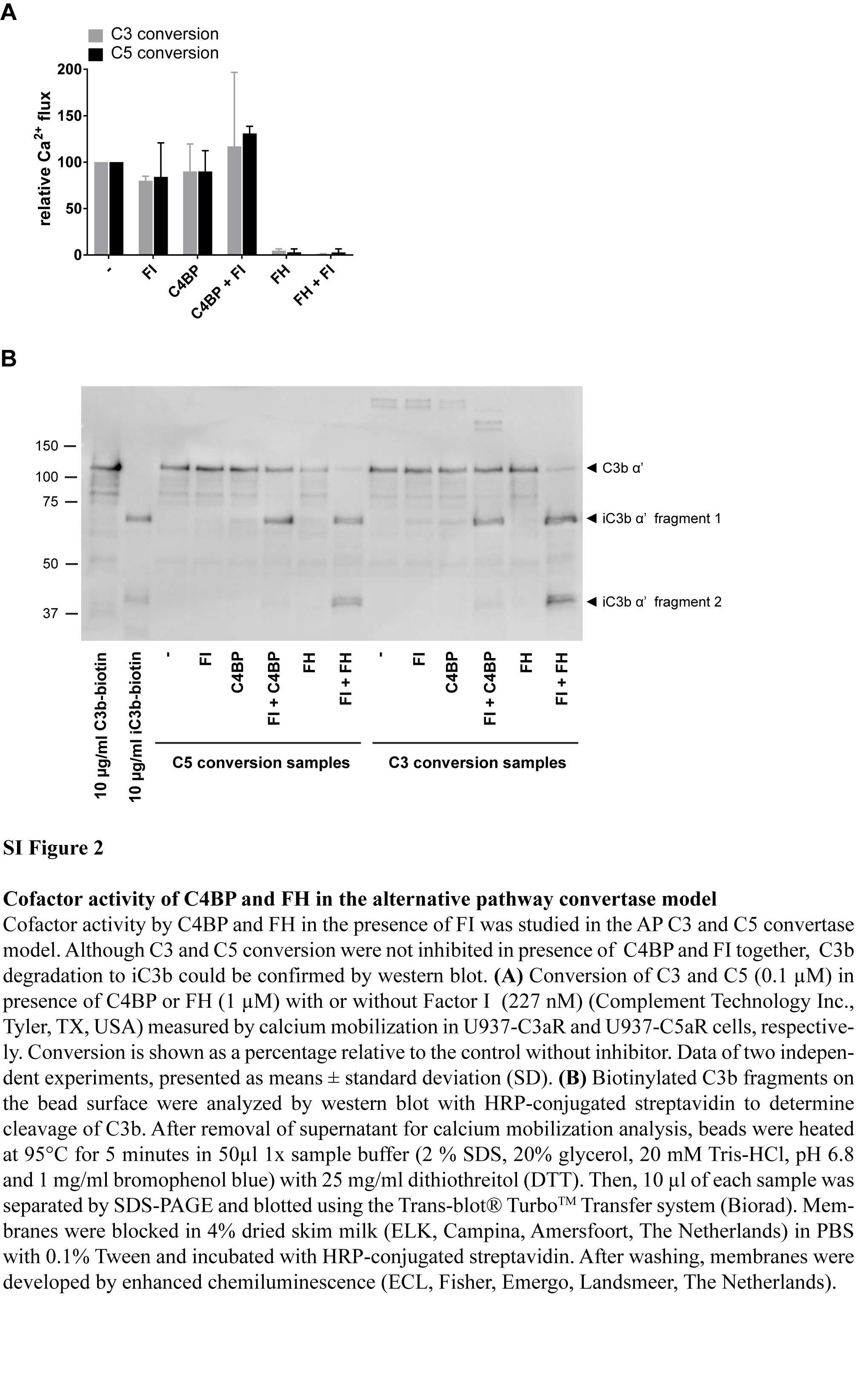

Supplement: Supplementary file 2 [file image_2.tif]
